# Modulatory Impacts of Multi-Strain Probiotics on Rabbits’ Growth, Nutrient Transporters, Tight Junctions and Immune System to Fight against *Listeria monocytogenes* Infection

**DOI:** 10.3390/ani12162082

**Published:** 2022-08-15

**Authors:** Marwa I. Abd El-Hamid, Doaa Ibrahim, Rehab I. Hamed, Heba H. Nossieur, Mariam Hassan Elbanna, Heba Baz, Ehab. M. Abd-Allah, Amal S. A. El Oksh, Ghada A. Ibrahim, Eman Khalifa, Tamer Ahmed Ismail, Naglaa F. S. Awad

**Affiliations:** 1 Department of Microbiology, Faculty of Veterinary Medicine, Zagazig University, Zagazig 44511, Egypt; 2 Department of Nutrition and Clinical Nutrition, Faculty of Veterinary Medicine, Zagazig 44511, Egypt; 3 Department of Poultry Diseases, Reference Laboratory for Quality Control of Poultry Production (RLQP), Animal Health Research Institute (AHRI), Zagazig Branch, Agriculture Research Center (ARC), Zagazig 44516, Egypt; 4 Department of Veterinary Public Health, Faculty of Veterinary Medicine, Zagazig University, Zagazig 44511, Egypt; 5 Veterinary Educational Hospital, Faculty of Veterinary Medicine, Zagazig University, Zagazig 44511, Egypt; 6 Department of Biotechnology, Reference Laboratory for Quality Control of Poultry Production (RLQP), Animal Health Research Institute (AHRI), Sharkia Branch, Agriculture Research Center (ARC), Zagazig 44511, Egypt; 7 Department of Bacteriology, Animal Health Research Institute (AHRI), Ismailia Branch, Agriculture Research Center (ARC), Ismailia 41522, Egypt; 8 Department of Microbiology, Faculty of Veterinary Medicine, Matrouh University, Matrouh 51511, Egypt; 9 Department of Clinical Laboratory Sciences, Turabah University College, Taif University, P.O. Box 11099, Taif 21944, Saudi Arabia; 10 Department of Avian and Rabbit Medicine, Faculty of Veterinary Medicine, Zagazig University, Zagazig 44519, Egypt

**Keywords:** multi-strain probiotics, cytokines, intestinal barrier, growth, *L. monocytogenes*, rabbits

## Abstract

**Simple Summary:**

Weaning is a crucial period associated with great stress and susceptibility to infection, implying adverse impacts on farmed rabbits’ production. Recently, probiotics have been provided as direct microbial feed supplements, which are considered the ideal antibiotic substitutes during pathogenic infections with an emphasis on promoting rabbits’ growth and modulating their immune functions. Therefore, our experiment was carried out to explore the efficacy of multi-strain probiotics (MSP) on rabbits’ growth, molecular aspects, such as nutrients transporters, cytokines, and intestinal integrity, and effectiveness against *Listeria monocytogenes* (*L. monocytogenes*) infection. Altogether, our findings proposed the beneficial consequences of MSP on rabbits’ growth, gut health, and immunity. After post-experimental infection of rabbits with *L. monocytogenes*, administration of MSP during the whole rearing period greatly reduced the detrimental impact of infection and consequently renovated efficient rabbits’ production.

**Abstract:**

Multi-strain probiotics (MSP) are considered innovative antibiotics’ substitutes supporting superior gut health and immunity of farmed rabbits. The promising roles of MSP on performance, intestinal immunity, integrity and transporters, and resistance against *Listeria monocytogenes* (*L. monocytogenes*) were evaluated. In the feeding trial, 220 rabbits were fed a control diet or diet supplemented with three MSP graded levels. At 60 days of age, rabbits were experimentally infected with *L. monocytogenes* and the positive control, enrofloxacin, prophylactic MSP (MSPP), and prophylactic and therapeutic MSP (MSPTT) groups were included. During the growing period, MSP at the level of 1 × 10^8^ CFU/kg diet (MSPIII) promoted the rabbits’ growth, upregulated the nutrient transporters and tight-junction-related genes, and modified cytokines expression. Supplementing MSPTT for *L. monocytogenes* experimentally-infected rabbits restored the impaired growth and intestinal barriers, reduced clinical signs of severity and mortalities, and attenuated the excessive inflammatory reactions. Notably, enrofloxacin decreased *L. monocytogenes* and beneficial microbial loads; unlike MSPTT, which decreased pathogenic bacterial loads and sustained the beneficial ones. Histopathological changes were greatly reduced in MSPTT, confirming its promising role in restricting *L. monocytogenes* translocation to different organs. Therefore, our results suggest the use of MSPTT as an alternative to antibiotics, thereby conferring protection for rabbits against *L. monocytogenes* infection.

## 1. Introduction

Rabbit breeding is becoming important due to the rapid growth rate, superior productive ability, and easily digestible and healthy meat of rabbits [1]. Weaning is the most stressful period associated with significant financial losses in rabbit husbandry as it can increase their susceptibility to numerous infections [2]. To overcome this period, strengthening the intestinal barrier and modifying the immune systems of rabbits are urgently needed, given that the intestinal barrier is the main site for the dynamic contact of enteric pathogens with the host [3]. It is well recognized that intestinal immune system development is triggered by commensal bacteria that inhibit the colonization of harmful bacteria and provide a protective barrier to resist the invasion of exogenous pathogens, thus preventing intestinal inflammation and ensuring intestinal integrity [4,5]. Any imbalance of microflora can lead to a change of pH and pathogen multiplication with harmful consequences for the animal’s health [6,7]. Moreover, several enteric pathogens can escape from commensal-mediated colonization resistance [8,9].

*Listeria monocytogenes* (*L. monocytogenes*) is one of the most devious pathogens associated with listeriosis, and is uniquely difficult to monitor [10]. Previous investigations showed that farmed rabbits are predisposed to *L. monocytogenes,* and their meat is a potential source of listerial foodborne pathogens [11,12]. The hardiness of *L. monocytogenes* against eradication is due to its capacity to adapt and persist in a variety of environments, its intracellular site, and the weak intracellular diffusion of some antibiotics, which makes it a serious ongoing concern in animal production [13,14]. Additionally, the virulence of *L. monocytogenes* arises from its ability for invasion, adhesion, and translocation across the intestinal barrier throughout the gastrointestinal phase of infection [15]. Therefore, restricting *L. monocytogenes* at the gastrointestinal stage of infection is the best way to restrict pathogen spread to the deeper tissues and avoid consequent lethality. Despite the fact that antibiotics are the treatment of choice for listeriosis, they can disrupt the gut microbiota and impair host defense immunity [16]. Besides, the progress of drug tolerant bacteria and antibiotic residues in the meat were serious drawbacks raised from the excessive use of antibiotics [17,18,19,20]. This in turn has facilitated the development of new antibiotics alternatives [21,22], such as probiotics, as a control strategy against listeria infection. Probiotics are living commensal micro-organisms that struggle with pathogens at the sites of adhesion, enhance nutrient utilization, enhance the epithelial immune response, augment microbial balance, restore epithelial barrier function, and, therefore, prevent infection and subsequent pathological lesions [23,24,25,26,27,28,29]. Probiotics belonging to either *Lactobacillus* or *Bacillus* species are commonly used in rabbits’ feed [24]. However, knowing what physiological changes the multi-strain probiotics (MSP) stimulate in healthy rabbits and their further efficacy under *L. monocytogenes* infection over long-term use is very limited. Moreover, the precise MSP mechanism of actions that assess their immune modulation and promising roles in preventing *L. monocytogenes* induced intestinal barrier dysfunction need further investigation. Keeping the above-mentioned facts in view, the present work was undertaken to investigate, for the first time, the effects of MSP—in comparison with enrofloxacin—on the growth performance and expression of nutrient transporters, cytokines, and tight-junction-protein-related genes and their roles in fighting *L. monocytogenes* experimental infection in rabbits.

## 2. Materials and Methods

### 2.1. Probiotics Preparation

Three probiotic bacterial strains, including *Lactobacillus acidophilus* NBIMCC 8242, *Bacillus subtilis* DSM 17,299 and *Enterococcus faecium* NBIMCC 8270, were mixed at equal ratios (1:1:1) before being added into the formulated feed. The probiotic strains were grown separately in de Man, Rogosa, and Sharpe broth (Oxoid, Cambridge, UK) at 37 °C for 48 h. After incubation, the cells were centrifuged and then spray-dried before being added to the rabbit feed.

### 2.2. Rabbits and Experimental Protocol

A total of 220 weaned New Zealand white male rabbits, aged approximately 30 days and having an 802.8 g average body weight, were obtained from commercial rabbit farms. To conduct the experiment, feeding, experimental infection, and clinical trials were formed. A four-week feeding trial was carried out to examine the effect of supplementing the basal diet with three graded levels of the three probiotic bacterial strains at equal ratios and to choose the appropriate and effective dose of the mixed probiotic strains to be used as a prophylactic and/or a novel therapeutic tool for *L. monocytogenes* experimental infection in the clinical trial.

#### 2.2.1. Feeding Trial

For this trial, 220 rabbits were randomly allocated into four equal experimental groups; each had 55 rabbits arranged in 5 replicates (11 rabbits each). The rabbits were offered the basal diet (control or untreated group) or fed experimental diets supplemented with a mixture of equal doses of *Lactobacillus acidophilus*, *Bacillus subtilis,* and *Enterococcus faecium* at graded levels—1 × 10^6^ (MSPI), 1 × 10^7^ (MSPII), and 1 × 10^8^ (MSPIII) colony forming units (CFU)/kg diet, respectively. All rabbits were kept in cages, fed pelleted diets, and provided free access to water and feed throughout the experimental rearing period. The diets were formulated in accordance with the nutrient recommendations for rabbits [30]. The ingredients and composition of the basal experimental diet are listed in Table 1.

#### 2.2.2. Experimental Infection and Clinical Trials

These trials were conducted on two experimental groups comprised of the control (rabbits fed the basal diet) and MSP (rabbits fed the experimental diets supplemented with the most effective dose of the mixed probiotic strains) groups. All rabbits were checked to be free from detectable *L. monocytogenes*. The experimental infection trial was conducted according to the protocol of an earlier study [31] using the field *L. monocytogenes* strain previously recovered from clinically-diseased rabbits. The used strain was revitalized in Listeria enrichment broth (Oxoid, Cambridge, UK) at 37 °C for 18 h under microaerophilic conditions. Subsequently, 0.1 mL of the enrichment broth was inoculated for 24 h at 37 °C onto a Listeria-selective agar base supplemented with Listeria-selective supplement (Oxoid, Cambridge, UK). The typical colonies of *L. monocytogenes* were confirmed by Gram’s stain and biochemical tests including lactose, sucrose and xylose fermentation, catalase, oxidase and urease according to FDA bacteriological analytical manual. The strain was further confirmed via umbrella shaped motility, hemolysis onto sheep blood agar (Oxoid, Cambridge, UK), CAMP test and molecular identification methods. The inoculum suspension was subsequently prepared to obtain a concentration of 10^7^ CFU/mL [32]. At 60 days of age, each rabbit in the two previous groups were orally administered with 1 mL of the prepared *L. monocytogenes* inoculum. Establishment of *L. monocytogenes* infection was verified by observing the characteristic clinical symptoms and post-mortem lesions of the sacrificed rabbits as well as bacteriological re-isolation and identification of the used strain. 

In the clinical trial, the above-mentioned experimental groups (*n* = 50/group) were further subdivided into 2 subgroups (*n* = 25/subgroup and each subgroup contained 5 replicates). Regarding the MSP group, the rabbits in the first subgroup received the MSP prophylactically for 30 days during the previous feeding trial (from 30 to 60 days of age) without further MSP supplementation after *L. monocytogenes* experimental infection (MSPP); meanwhile, those in the second subgroup were offered the same MSP for an additional 30 days after *L. monocytogenes* experimental infection (from 30 to 90 days of age, MSPTT). Concerning the control group, the rabbits in the first subgroup were kept infected only and did not receive any treatment, but those in the second subgroup were treated with commercial enrofloxacin hydrochloride (ENR) at a dose of 40 mg/kg for 7 consecutive days, as was recommended by the producer [32]. The treatment program with ENR antibiotic was applied when the rabbits presented the clinical manifestations associated with listeriosis to support successful antibiotic treatment of *L. monocytogenes* infection.

### 2.3. Growth Performance Traits and Clinical Examination

The growth performance parameters of rabbits, in each replicate, were evaluated at the end of both the growing and finishing periods (60 and 90 days of age, respectively). The feed intake (FI) and body weight gain (BWG) were verified and then the feed conversion ratio (FCR) was estimated as following: amount of consumed feed (g)/BWG (g) [33,34]. Additionally, clinical signs and post-mortem (PM) findings were recorded. Moreover, the mortality rates of rabbits were calculated during the course of the experimental period.

### 2.4. Samples Collection

At 60 (before experimental infection) and 90 (4 weeks after *L. monocytogenes* experimental infection) days of age, 5 experimental rabbits were randomly selected and sacrificed by cervical dislocation. The slaughtering and dissection of rabbits were performed according to the recommendations of the World Rabbit Science Association [35]. The cecal and ileal contents were collected for further quantitative analysis of intestinal microbiota. The collected cecal and ileal contents were stored at −80 °C until analyzed with commercial DNA extraction kits. Moreover, slaughtered rabbits were skinned, eviscerated, and tissues from the jejunum and cecum were then collected and stored in RNAlater^®^ (Sigma Aldrich, St. Louis, MO, USA) for subsequent mRNA expression assays. At 7 and 14 days post-experimental infection, *L. monocytogenes* counts were enumerated in the cecal contents. Finally, samples of liver, brain, and spleen tissues were harvested and fixed in 10% neutral buffered formalin for histopathological examination at 14 days post-infection according to [36,37].

### 2.5. Investigated Parameters

#### 2.5.1. Quantitative DNA-Based Analysis of Intestinal Bacterial Populations

Total DNA was extracted from the cecal and ileal contents using QIAamp Fast DNA Stool Mini (Qiagen, Hilden, Germany). The extracted DNA quality and concentration were assessed by a Thermo Scientific NanoDrop 2000 spectrophotometer (Thermo Fisher Scientific Inc., Waltham, MA, USA). Ultimately, the purified DNA samples were stored at −80 °C for posterior quantitative PCR analysis. Real-time PCR (RT-PCR) assays were performed to calculate the populations of some intestinal microbial species, including total bacteria, and *Lactobacillus*, *Bifidobacterium*, *Enterobacteriaceae,* and *L. monocytogenes* strains using a Stratagene MX3005P quantitative PCR machine. The sequences of the primers targeting the specific bacterial genes are shown in Table 2. The PCR amplification assays were carried out, in triplicate, in a reaction volume of 25 μL containing 12.5 μL of SYBR Green PCR Master Mix (Qiagen, Hilden, Germany), 1 μL of of each primer (10 mM), 2 μL of target genomic DNA, and 8.5 μL of sterile PCR-grade water. Standard curves were prepared with ten-fold serial dilutions of genomic DNA isolated from pure bacterial cultures. The standard calibration curves were then generated by plotting the threshold cycle (Ct) values versus the bacterial DNA copy numbers. The bacterial concentrations in each DNA sample were measured using the generated standard curves in terms of log_10_ CFU/gram of the cecal and ileal contents.

#### 2.5.2. Expression Profiling by Reverse Transcription Quantitative Real-Time PCR (qRT-PCR) Assays

The extracted total RNA from frozen jejunum and cecum tissues was done via QIAamp RNeasy Mini kit (Qiagen, Hilden, Germany) in compliance with the manufacturer’s instructions. RNA concentration and purity were assessed through a Nano Drop 2000 spectrophotometer (Thermo Scientific Inc., Waltham, MA, USA). The expression analysis was determined by one-step qRT-PCR assays using 2× QuantiTect SYBR Green RT-PCR Kit (Qiagen, Hilden, Germany). The mRNA expression levels of genes encoding nutrient transporters including sodium–glucose co-transporter-1 (SGLT-1), glucose transporter-2 (GLUT-2), cationic amino acid transporter-1 (CAT-1), and fatty acid-binding protein-2 (FABP-2); tight junction proteins (TJP) comprising claudins-1 (CLDN-1), junctional adhesion molecule-2 (JAM-2), occludin, and mucin-2 (MUC-2); and cytokines including interleukin-6 (IL-6), IL-8, IL-1β, IL-10, transforming growth factor-beta-1 (TGF-β1), and tumor necrosis factor-alpha (TNF-α) were determined at 60 and 90 days of age. Moreover, the mRNA expression level of gene encoding interferon gamma (IFNγ) was detected at 90 days of age only. All qRT-PCR reactions were run, in triplicate, on a Stratagene Mx3005P real-time thermal cycler (Agilent Technologies, Inc., Santa Clara, CA, USA). The transcript levels of the genes of interest were normalized to the expression of the housekeeping gene, glyceraldehyde 3-phosphate dehydrogenase (*GAPDH*), as a calibrator. All the investigated target genes and the sequences of their appropriate primers are characterized in Table 2. After the qRT-PCR reactions were completed, the specificity of the PCR amplifications and the purity of qPCR products were verified by post-PCR melting curves analyses. The relative changes in gene expression levels were analyzed using the 2^−ΔΔCt^ method [38].

**Table 2:** Primer sequences of target genes analyzed in quantitative real-time PCR assays.

Encoding Gene	Primer Sequence (5′-3′)	Accession No./Reference
Nutrient transporters		
*SGLT-1*	F:GATTTCCCGTATGATTACCGAG R:AAGAGGGAGACAACCACAACG	NM_001101692.1
*GLUT-2*	F-CAGAGGCACTGTCCACCACC R:TGTCTCCAAGCCACCCACC	NM_001277382.1
*CAT-1*	F:CCAGTCTATTAGGTTCCATGTTCC R: CGATTATTGGCGTTTTGGTC	XM_002721425.3
*FABP-2*	F: GTGGGGTTTTCCCTTTTGC R: CGCACTTTGGCCTTCACC	XM_002722970
Tight junction proteins		
*MUC-2*	F:TATACCGCAAGCAGCCAGGT R:GCAAGCAGGACACAGACCAG	L41544.1
*JAM-2*	F:ATATCGCAGGTGTCCTGGAA R: GAGCATAGCACACGCCAAG	XM_017346699
*CLDN-1*	F: GGAGCAAAAGATGCGGATGG R: AATTGACAGGGGTCAAAGGGT	NM_001089316.1
*occludin*	F: GCAAGAGGCCGTATCCAGAG R: AGTCCGTCTCGTAGTGGTCT	XM_008262320.1
Cytokines		
*IL-6*	F:GCCAACCCTACAACAAGA R:AGAGCCACAACGACTGAC	NC_013678
*IL-8*	F:CTCTCTTGGCAACCTTCCTG R:TTGCACAGTGAGGTCCACTC	KT216053.1
*IL-10*	F:AAAAGCTAAAAGCCCCAGGA R:CGGGAGCTGAGGTATCAGAG	NM001082045.1
*IL-1β*	F:TTCCGGATGTATCTCGAGCA R:GTGGATCGTGGTCGTCTTCA	NC_013670
*TNF-α*	F:CTGCACTTCAGGGTGATCG R:CTACGTGGGCTAGAGGCTTG	XM_008262537.2
*IFNγ*	F:TTCTTCAGCCTCACTCTCTCC R:TGTTGTCACTCTCCTCTTTCC	NM_001081991.1
*TGF* *-β1*	F: CAGTGGAAAGACCCCACATCTC R: GACGCAGGCAGCAATTATCC	NM_001082660
House keeping		
*GAPDH*	F:TGTTTGTGATGGGCGTGAA R:CCTCCACAATGCCGAAGT	NC_013676.1
*Listeria monocytogenes*		
*16S rRNA*	F: CCTTTGACCACTCTGGAGACAGAGC R: AAGGAGGTGATCCAACCGCACCTTC	[39]
Total bacteria		
* 16S rRNA *	F: CGGCAACGAGCGCAACCC R: CCATTGTAGCACGTGTGTAGCC	[40]
Genus *Lactobacillus*		
* 16S rRNA *	F: AGCAGTAGGGAATCTTCCA R: CACCGCTACACATGGAG	[40]
Genus *Bifidobacterium*		
* 16S rRNA *	F: TCGCGTCYGGTGTGAAAG R: CCACATCCAGCRTCCAC	[40]
*Enterobacteriaceae*		
* 16S rRNA *	F: CATTGACGTTACCCGCAGAAGAAGC R: CTCTACGAGACTCAAGCTTGC	[40]

*SGLT-1*: sodium–glucose co-transporter-1; *GLUT-2*: glucose transporter-2; *CAT-1*: cationic amino acid transporter-1; *FABP-2*: fatty acid-binding protein-2; *MUC-2*: mucin-2; *JAM-2*: junctional adhesion molecule-2; *CLDN-1:* claudins-1; *IL*: interleukin; *TNF-α:* tumor necrosis factor-alpha; *IFNγ*: interferon gamma; *TGF-β1*: transforming growth factor-beta-1; *GAPDH*: glyceraldehyde 3-phosphate dehydrogenase and *16S rRNA*: 16S ribosomal RNA.

#### 2.5.3. Histomorphological Examination

The fixed liver, brain, and spleen tissues were cut to size, washed with fresh water, dehydrated in ascending grades of absolute ethanol, transparentized in xylene, and ultimately impregnated in paraffin wax. Thin sections (5 μm in thickness) of paraffin-embedded tissues were stained with eosin and hematoxylin and examined under the light microscope equipped with a computerized digital camera [37,41,42]. Stained slides were analyzed, and the lesions were accordingly detected and documented.

### 2.6. Statistical Analysis

The results were analyzed by the GLM procedure of SPSS and a subsequent Tukey’s post-hoc test was used to estimate the statistically significant differences among the treatment groups. Homogeneity and normality among our groups were evaluated through Levene’s and Shapiro–Wilk’s tests, correspondingly. The achieved data were conveyed as standard error of mean (SEM). For all tests, the significance was determined at *p* < 0.05.

## 3. Results

### 3.1. Growth Performance and Clinical Observations

The results regarding the growth performance attributes of rabbits are shown in Table 3 and Table 4. At the end of the growing period, the BWG of rabbits were significantly (*p* < 0.05) increased with increasing levels of MSP. Moreover, supplementation with MSP at a level of 1 × 10^8^ CFU/kg displayed the most significant (*p* < 0.05) improvement in rabbits’ FCR (Table 3). At the end of finishing period (90 d), infecting rabbits with *L. monocytogenes* significantly (*p* < 0.05) decreased BWG and impaired FCR. Interestingly, this impaired growth rate and FCR were improved in the probiotics- or enrofloxacin-treated groups. Meanwhile, rabbits receiving MSP (prophylactic and therapeutic) had the highest significant (*p* < 0.05) BWG and improved FCR. The experimentally-infected rabbits showed various clinical signs in the form of depression, anorexia and diarrhea, and generalized septicemia in PM examination of freshly dead rabbits. Notably, MSP over the whole rearing period (MSPTT) reduced the severity of both clinical signs and PM lesions. Moreover, the mortality percentages were significantly (*p* < 0.05) reduced, especially after administration of MSP during the whole rearing period when compared with the positive control group (24 vs. 6%) (Table 4).

### 3.2. Microbial Populations of Intestinal Contents

**Figure 1:**
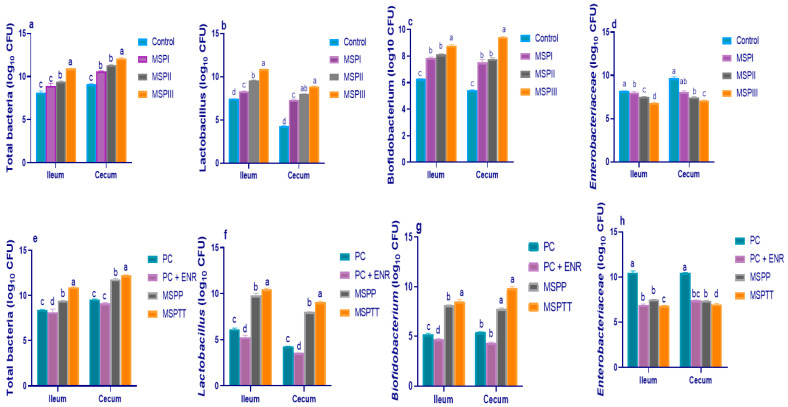
Effects of various levels of multi-strain probiotics (**a**–**d**) and multi-strain probiotics either used prophylactically and/or therapeutically and enrofloxacin treatment (**e**–**h**) on total aerobic bacterial, *Lactobacillus, Bifidobacterium,* and *Enterobacteriaceae* populations (log_10_ CFU) in the ileum and cecum of rabbits at 60 and 90 d of age, respectively. MSP: multi-strain probiotics including *Lactobacillus acidophilus* NBIMCC 8242, *Bacillus subtilis* DSM 17,299, and *Enterococcus faecium* NBIMCC 8270. Control: rabbits fed a basal diet; MSPI, II and III: rabbits fed a basal diet supplemented with MSP at the levels of 1 × 10^6^, 1 × 10^7^, and 1 × 10^8^ CFU/kg, respectively. PC: positive control (rabbits fed a basal diet and experimentally infected with *L. monocytogenes*); PC + ENR: rabbits fed a basal diet, experimentally infected with *L. monocytogenes,* and treated with enrofloxacin; MSPP: rabbits fed a basal diet supplemented with MSP at the level of 1 × 10^8^ CFU/kg from 30 to 60 days of age (prophylactic) and experimentally infected with *L. monocytogenes*; MSPTT: rabbits fed a basal diet supplemented with MSP at the level of 1 × 10^8^ CFU/kg from 30 to 90 days of age (prophylactic and therapeutic) and experimentally infected with *L. monocytogenes*. Rabbits were orally administered with *L. monocytogenes* at the concentration of 10^7^ CFU/mL at 60 days of age. Values are means with their SE in bars. ^a–d^: Means inside the same column having various superscripts are significantly different at *p* < 0.05.

At 60 days of age (before *L. monocytogenes* experimental infection), MSP supplementation at various levels resulted in a differential increase in the abundance of the total aerobic bacterial (Figure 1a), *Lactobacillus* (Figure 1b), and *Bifidobacterium* (Figure 1c) populations and decreased *Enterobacteriaceae* counts (Figure 1d) compared to the control group. Furthermore, compared with the control group, supplementing the rabbit diet with MSP at 1 × 10^8^ CFU/kg remarkably elevated (*p* < 0.05) the abundance of *Lactobacillus* and *Bifidobacterium* counts by 3.45 and 2.51 CFU/g of the ileal contents and 4.65 and 4 CFU/g of the cecal contents, respectively. In contrast, *Enterobacteriaceae* counts were notably decreased when increasing the levels of MSP in rabbits’ diets. At 90 days of age, rabbits experimentally infected with *L. monocytogenes* exhibited the highest (*p* < 0.05) colonization of harmful *Enterobacteriaceae* and the lowest (*p* < 0.05) populations of beneficial ones (i.e., *Lactobacillus* and *Bifidobacterium* species) in the ileum and cecum. Notably, enrofloxacin-treated rabbits exhibited the lowest (*p* < 0.05) ileal and cecal total bacteria (Figure 1e), and *Lactobacillus* (Figure 1f) and *Bifidobacterium* (Figure 1g) populations. Meanwhile, the cecum and ileum of experimentally-infected rabbits that received MSP had lower *Enterobacteriaceae* (Figure 1h) counts and higher *Lactobacillus* and *Bifidobacterium* populations, with prominent beneficial effects in rabbit groups that received MSP during the whole rearing period (prophylactic and therapeutic, MSPTT).

### 3.3. Gene Expression Analysis

**Figure 2:**
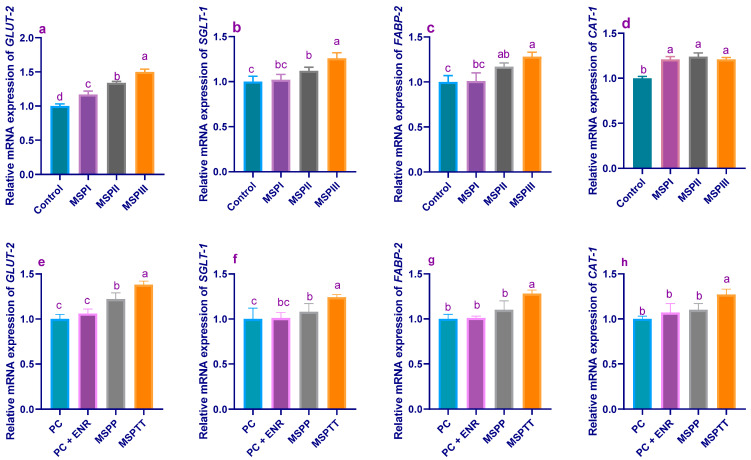
Levels of glucose transporter-2 (*GLUT-2*), sodium–glucose co-transporter-1 (*SGLT-1*), fatty acid-binding protein-2 (*FABP-2*), and cationic amino acid transporter-1 (*CAT-1*) mRNA expression in rabbits fed different levels of multi-strain probiotics at the end of the growing period (60 d of age; (**a**–**d**)) and those experimentally infected with *L. monocytogenes* and supplemented with multi-strain probiotics either used prophylactically and/or therapeutically and enrofloxacin treatment (at 90 d of age; (**e**–**h**)). MSP: multi-strain probiotics including *Lactobacillus acidophilus* NBIMCC 8242, *Bacillus subtilis* DSM 17,299, and *Enterococcus faecium* NBIMCC 8270. Control: rabbits fed a basal diet; MSPI, II and III: rabbits fed a basal diet supplemented with MSP at the levels of 1 × 10^6^, 1 × 10^7^, and 1 × 10^8^ CFU/kg, respectively. PC: positive control (rabbits fed a basal diet and experimentally infected with *L. monocytogenes*); PC + ENR: rabbits fed a basal diet, experimentally infected with *L. monocytogenes,* and treated with enrofloxacin; MSPP: rabbits fed a basal diet supplemented with MSP at the level of 1 × 10^8^ CFU/kg from 30 to 60 days of age (prophylactic) and experimentally infected with *L. monocytogenes*; MSPTT: rabbits fed a basal diet supplemented with MSP at the level of 1 × 10^8^ CFU/kg from 30 to 90 days of age (prophylactic and therapeutic) and experimentally infected with *L. monocytogenes*. Rabbits were orally administered with *L. monocytogenes* at the concentration of 10^7^ CFU/mL at 60 days of age. Values are means with their SE in bars. ^a–d^: Means inside the same column having different superscripts are significantly different at *p* < 0.05.

The results of a gene expression analysis of nutrient-transporter-related genes analyzed by RT-PCR are illustrated in Figure 2. Before *L. monocytogenes* experimental infection (60 d of age), the data indicated that *GLUT-2* gene expression levels were significantly (*p* < 0.05) increased when increasing the MSP levels (Figure 2a). Additionally, the mRNA expression levels of *SGLT-1* (Figure 2b) and *FABP-2* (Figure 2c) genes reached their peaks in rabbits supplemented with MSP at the level of 1 × 10^8^ CFU/kg (1.26- and 1.28-fold, respectively). Moreover, the transcriptional levels of *CAT-1* genes (Figure 2d) were increased post-MSP supplementation, unlike the control group—with no significant differences detected among different MSP levels. At 90 d of age, expression analysis displayed significant (*p* < 0.05) upregulation of *GLUT-2* genes (Figure 2e) in rabbits fed MSP-supplemented diets over the whole rearing period (MSPTT), followed by those fed MSP as a prophylactic, unlike the experimentally-infected (PC) group. Additionally, rabbits that received MSP during the whole rearing period exhibited the highest (*p* < 0.05) upregulation in *SGLT-1* (Figure 2f), *FABP-2* (Figure 2g), and *CAT-1* (Figure 2h) gene expression (1.24-, 1.28-, and 1.27-fold, respectively).

**Figure 3:**
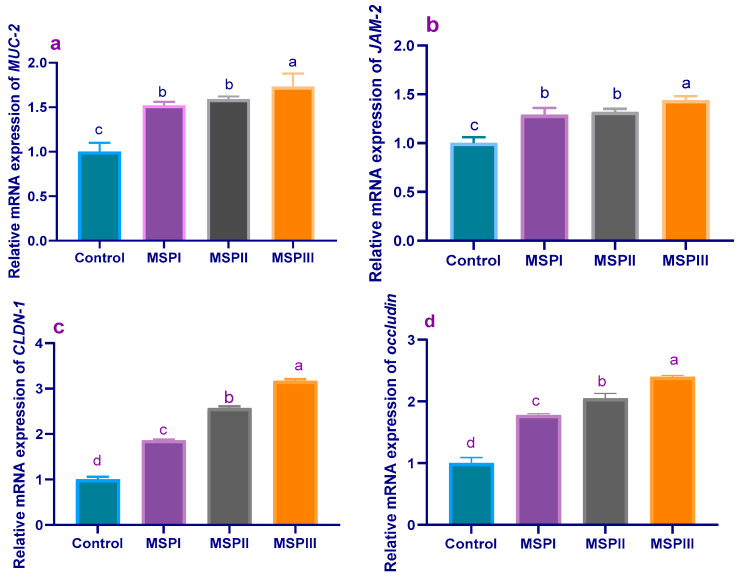
Levels of mucin-2 (*MUC-2*, (**a**)), junctional adhesion molecule-2 (*JAM-2*, (**b**)), claudins-1 (*CLDN-1*, (**c**)), and occludin (**d**) mRNA expression in rabbits fed different levels of multi-strain probiotics at the end of the growing period (60 d of age). MSP: multi-strain probiotics including *Lactobacillus acidophilus* NBIMCC 8242, *Bacillus subtilis* DSM 17,299, and *Enterococcus faecium* NBIMCC 8270. Control: rabbits fed a basal diet; MSPI, II and III: rabbits fed a basal diet supplemented with MSP at the levels of 1 × 10^6^, 1 × 10^7^, and 1 × 10^8^ CFU/kg, respectively. Values are means with their SE in bars. ^a–d^: Means inside the same column having various superscripts are significantly different at *p* < 0.05.

**Figure 4:**
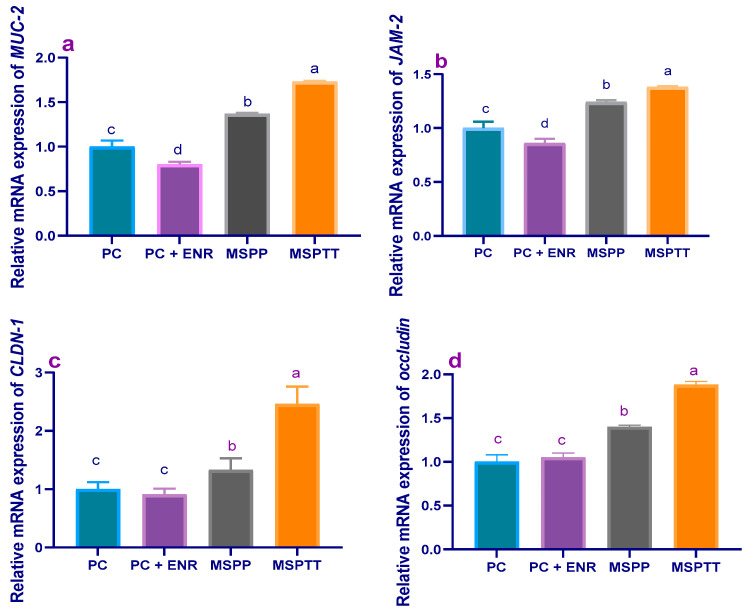
Levels of mucin-2 (*MUC-2*, (**a**)), junctional adhesion molecule-2 (*JAM-2*, (**b**)), claudins-1 (*CLDN-1*, (**c**)), and occludin (**d**) mRNA expression in rabbits experimentally infected with *L. monocytogenes* and supplemented with multi-strain probiotics either used prophylactically and/or therapeutically and enrofloxacin treatment (at 90 d of age). MSP: multi-strain probiotics including *Lactobacillus acidophilus* NBIMCC 8242, *Bacillus subtilis* DSM 17,299, and *Enterococcus faecium* NBIMCC 8270. PC: positive control (rabbits fed a basal diet and experimentally infected with *L. monocytogenes*); PC + ENR: rabbits fed a basal diet, experimentally infected with *L. monocytogenes,* and treated with enrofloxacin; MSPP: rabbits fed a basal diet supplemented with MSP at the level of 1 × 10^8^ CFU/kg from 30 to 60 days of age and experimentally infected with *L. monocytogenes*; MSPTT: rabbits fed a basal diet supplemented with MSP at the level of 1 × 10^8^ CFU/kg from 30 to 90 days of age and experimentally infected with *L. monocytogenes*. Rabbits were orally administered with *L. monocytogenes* at the concentration of 10^7^ CFU/mL at 60 days of age. Values are means with their SE in bars. ^a–d^: Means inside the same column having superscripts are significantly different at *p* < 0.05.

The results of a gene expression analysis of tight-junction-protein-related genes are illustrated in Figure 3 and Figure 4. Before experimental infection (60 d of age), the expression levels of *MUC-2* (Figure 3a) and *JAM-2* (Figure 3b) were prominently upregulated (*p* < 0.05) in groups fed MSP at a level of 1 × 10^8^ CFU/kg when compared with the control group (1.73- and 1.44-fold, respectively). Moreover, *CLDN-1* (Figure 3c) and occludin (Figure 3d) transcriptional levels were increased (*p* < 0.05) post-supplementation with MSP in a dose-dependent manner. At 90 d of age, the maximum expression levels of *MUC-2* (Figure 4a) and *JAM-2* (Figure 4b) were observed in rabbits fed MSP for either 30 or 60 days; meanwhile, their expressions were significantly (*p* < 0.05) impaired in rabbits treated with enrofloxacin. Of note, the expression results showed significant (*p* < 0.05) upregulation of *CLDN-1*(Figure 4c) and occludin (Figure 4d) genes in rabbits supplemented with MSP during the whole rearing period (MSPTT), followed by those fed MSP as a prophylactic (MSPP)—in comparison with the experimentally-infected (PC) group.

**Figure 5:**
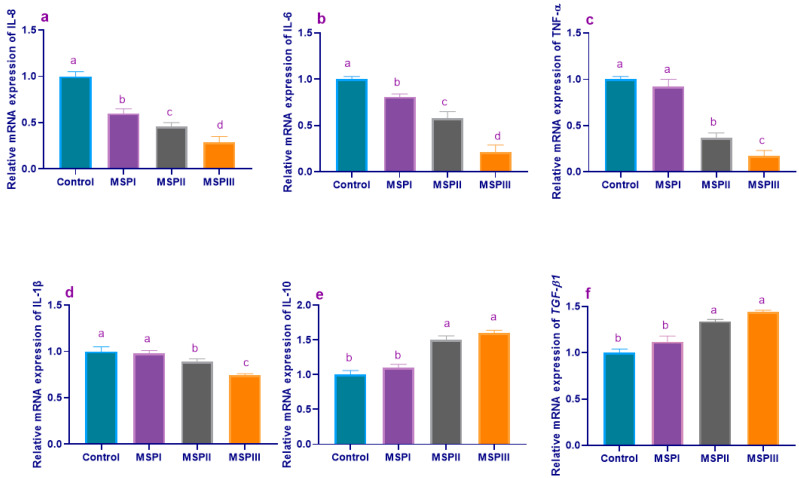
Levels of interleukin-8 (*IL-8*, (**a**)), interleukin-6 (*IL-6*, (**b**)), tumor necrosis factor-alpha (*TNF-α*, (**c**)), interleukin-1-beta (*IL-1β*, (**d**)), interleukin-10 (*IL-10*, (**e**)), and transforming growth factor-beta-1 (*TGF-β1*, (**f**)) mRNA expression in rabbits fed different levels of multi-strain probiotics at the end of growing period (60 d of age). MSP: multi-strain probiotics including *Lactobacillus acidophilus* NBIMCC 8242, *Bacillus subtilis* DSM 17,299, and *Enterococcus faecium* NBIMCC 8270. Control: rabbits fed a basal diet; MSPI, II and III: rabbits fed a basal diet supplemented with MSP at the levels of 1 × 10^6^, 1 × 10^7^, and 1 × 10^8^ CFU/kg, respectively. Values are means with their SE in bars. ^a–d^: Means inside the same column having different superscripts are significantly different at *p* < 0.05.

**Figure 6:**
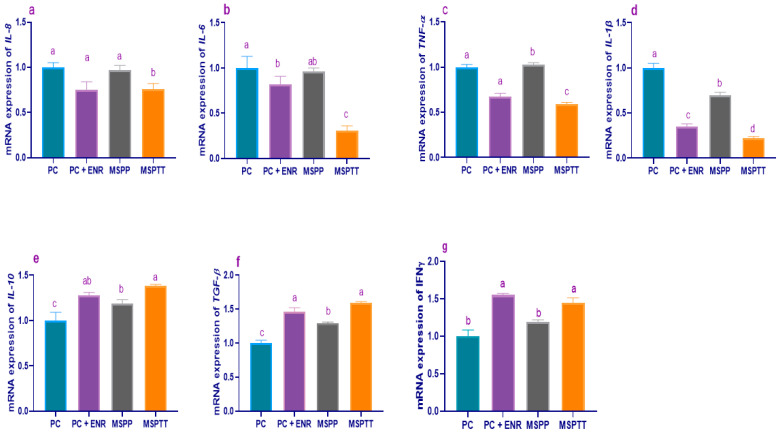
Levels of interleukin-8 (*IL-8*, (**a**)), interleukin-6 (*IL-6,* (**b**)), tumor necrosis factor-alpha (*TNF-α,* (**c**)), interleukin-1-beta (*IL-1β,* (**d**)); interleukin-10 (*IL-10,* (**e**)), transforming growth factor-beta-1 (*TGF-β1,* (**f**)), and interferon gamma (*IFNγ,* (**g**)), mRNA expression in rabbits experimentally infected with *L. monocytogenes* and supplemented with multi-strain probiotics either used prophylactically and/or therapeutically and enrofloxacin treatment (at 90 d of age). MSP: multi-strain probiotics including *Lactobacillus acidophilus* NBIMCC 8242, *Bacillus subtilis* DSM 17,299, and *Enterococcus faecium* NBIMCC 8270. PC: positive control (rabbits fed a basal diet and experimentally infected with *L. monocytogenes*); PC+ENR: rabbits fed a basal diet, experimentally infected with *L. monocytogenes,* and treated with enrofloxacin; MSPP: rabbits fed a basal diet supplemented with MSP at the level of 1 × 10^8^ CFU/kg from 30 to 60 days of age and experimentally infected with *L. monocytogenes*; MSPTT: rabbits fed a basal diet supplemented with MSP at the level of 1 × 10^8^ CFU/kg from 30 to 90 days of age and experimentally infected with *L. monocytogenes*. Rabbits were orally administered with *L. monocytogenes* at the concentration of 10^7^ CFU/mL at 60 days of age Values are means with their SE in bars. ^a–d^: Means inside the same column having various superscripts are significantly different at *p* < 0.05.

The results of a gene expression analysis of cytokine-related genes are illustrated in Figure 5 and Figure 6. Before experimental infection with *L. monocytogenes* (60 d of age), the relative expression levels of *IL-8* (Figure 5a) and *IL-6* (Figure 5b) genes were significantly (*p* < 0.05) decreased with increasing the levels of dietary MSP, while *TNF-α* (Figure 5c) and *IL-1β* (Figure 5d) expression levels were significantly (*p* < 0.05) decreased in rabbits fed MSP at levels of 1 × 10^7^ and 1 × 10^8^ CFU/kg unlike the control group. Moreover, *IL-10* (Figure 5e) and *TGF-β1* (Figure 5f) gene expression levels were significantly (*p* < 0.05) upregulated, especially with higher MSP supplementation levels. At 90 d of age, other remarkable findings emerged from the data analyses were the prominant decreases in the expression levels of the *IL-8* (Figure 6a), *IL-6* (Figure 6b), *TNF-α* (Figure 6c), and *IL-1β* (Figure 6d) genes in rabbits fed MSP over the whole rearing period (MSPTT) and those treated with enrofloxacin. Additionally, the enrofloxacin- and prophylactic and therapeutic MSP (MSPTT) treated groups, followed by prophylactic MSP (MSPP), displayed higher (*p* < 0.05) *IL-10* (Figure 6e) and *TGF-β1* (Figure 6f) transcriptional levels compared with the experimentally-infected group. Moreover, the most prominent (*p* < 0.05) upregulation of the *IFNγ* gene (Figure 6g) was detected in rabbits fed MSP during the whole rearing period (MSPTT) and those treated with enrofloxacin (1.45- and 1.55-fold, respectively).

### 3.4. Quantification of L. monocytogenes

**Figure 7:**
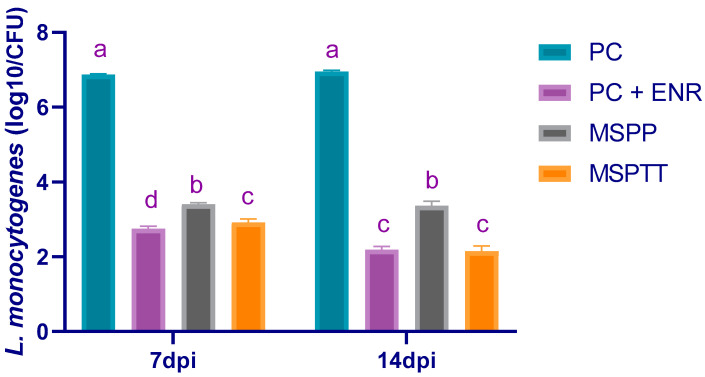
Quantification of cecal *L. monocytogenes* populations in response to multi-strain probiotics (either prophylactically and/or therapeutically) supplementation and enrofloxacin (ENR) at 7- and 14-days post-infection (dpi), as measured by real-time PCR assay. MSP: multi-strain probiotics including *Lactobacillus acidophilus* NBIMCC 8242, *Bacillus subtilis* DSM 17,299, and *Enterococcus faecium* NBIMCC 8270. PC: positive control (rabbits fed a basal diet and experimentally infected with *L. monocytogenes*); PC+ENR: rabbits fed a basal diet, experimentally infected with *L. monocytogenes,* and treated with enrofloxacin; MSPP: rabbits fed a basal diet supplemented with MSP at the level of 1 × 10^8^ CFU/kg from 30 to 60 days of age and experimentally infected with *L. monocytogenes*; MSPTT: rabbits fed a basal diet supplemented with MSP at the level of 1 × 10^8^ CFU/kg from 30 to 90 days of age and experimentally infected with *L. monocytogenes*. Rabbits were orally administered with *L. monocytogenes* at the concentration of 10^7^ CFU/mL at 60 days of age Values are means with their SE in bars. ^a–d^: Means inside the same column having different superscripts are significantly different at *p* < 0.05.

At 7 days post-experimental infection, significantly (*p* < 0.05) lower log_10_ copies of *L. monocytogenes* populations were found in the cecal contents of rabbits treated with enrofloxacin, followed by those fed MSP during the whole rearing period (MSPTT; 2.75 and 2.92 log_10_ CFU/g, respectively) (Figure 7). Another remarkable observation that emerged from the data analyses was that *L. monocytogenes* counts reached their lowest levels in rabbits either treated with enrofloxacin or those that were supplemented with MSP during the whole rearing period (MSPTT) at 14 days post-infection (Figure 7).

### 3.5. Histopathological Alterations

**Figure 8:**
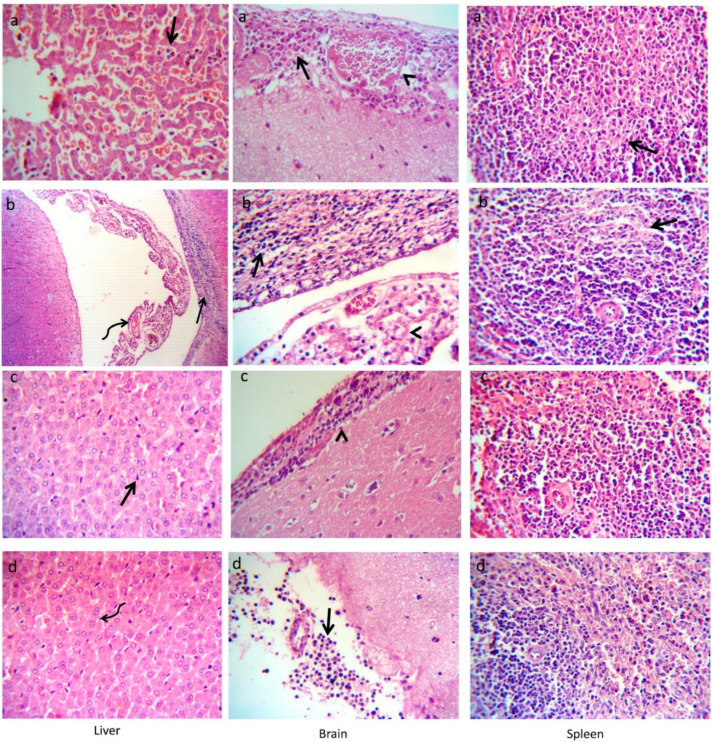
Histopathological alterations of rabbits’ liver, brain, and spleen at 14-days post *L. monocytogenes* experimental infection. (**a**) PC: positive control (rabbits fed a basal diet and experimentally infected with *L. monocytogenes*). Neutrophilic infiltrations within sinusoids (arrow) in hepatic parenchyma. Meningeal congestion (arrowhead) and leukocytic infiltrations, mainly neutrophils (arrow), in the brain tissue. Shrinkage of some lymphoid follicles and necrotic changes of some lymphoid elements (arrow) in the spleen. (**b**) PC+ENR: rabbits fed a basal diet, experimentally infected with *L. monocytogenes,* and treated with enrofloxacin. Thrombus (curved arrow) in the blood vessels and neutrophilic infiltrations within sinusoids (arrow) in hepatic parenchyma. Meningeal congestion (arrowhead) and leukocytic infiltrations, mainly neutrophils (arrow), in the brain tissue. Shrinkage of some lymphoid follicles and necrotic changes of some lymphoid elements (arrow) within the spleen. (**c**) MSPP: rabbits fed a basal diet supplemented with MSP at the level of 1 × 10^8^ CFU/kg from 30 to 60 days of age and experimentally infected with *L. monocytogenes*. Normal hepatic cords, and blood vessels with prominent kupfurr cells (arrow). Brain with periventricular inflammatory cells infiltration (arrowhead). Spleen with mild to moderate proliferation of white pulp lymphoid population. (**d**) MSPTT: rabbits fed a basal diet supplemented with MSP at the level of 1 × 10^8^ CFU/kg from 30 to 90 days of age and experimentally infected with *L. monocytogenes*. Normal hepatic parenchyma (curved arrow) and normal cerebral tissue with the presence of a number of round cells infiltrating the meninges (arrow). Preserved white pulp and red pulp with moderate infiltration of red pulp by mature and immature lymphocytes and other inflammatory cells. Rabbits were orally administered with *L. monocytogenes* at the concentration of 10^7^ CFU/mL at 60 days of age. Magnification power was assessed at 400×.

At 14-days post-infection, the liver of *L. monocytogenes* experimentally-infected rabbits (positive control group) showed congestion of the hepatic blood vessels and sinusoids. Moreover, thrombus was formed within blood vessels and neutrophilic infiltrations were seen within sinusoids. The brain showed suppurative meningitis, which is characterized by an increase in the thickness of the meninges due to meningeal congestion and leukocytic infiltrations, mainly neutrophils. The spleen showed shrinkage of some lymphoid follicles and necrotic changes of some lymphoid elements besides dilated splenic sinusoids (Figure 8a). The histopathological findings of rabbits experimentally infected with *L. monocytogenes* and treated with enrofloxacin are shown in Figure 8b. The liver showed a preserved lobular pattern, cord arrangement, central veins, and portal triads structures with the presence of thrombus in the blood vessels and neutrophilic infiltrations within sinusoids in the hepatic parenchyma. The brain tissue showed abundant cellular and karyorrhectic debris of inflammatory cells within periventricular tissue with ventricular congestion, edema, and necrosis of some choroid plexus epithelium. The examined sections of the spleen showed depleted lymphocytes within some white pulp. Additionally, the histopathological outcomes of the liver, brain, and spleen of rabbits that received a diet supplemented with MSP as a prophylactic and experimentally infected with *L. monocytogenes* (MSPP) are shown in Figure 8c. The examined liver section tissues showed normal hepatic cords and blood vessels with prominent kupffur cells. Most of the cerebral parenchyma had normal structures. However, the periventricular inflammatory cells were seen. The spleen revealed mild to moderate proliferation of the white pulp lymphoid population. The red pulp showed congestion of splenic blood vessels and it was infiltrated by a large number of mature and immature lymphocytes and proliferated macrophages. The histopathological alterations of rabbits fed MSP over the whole rearing period (MSPTT) and experimentally infected with *L. monocytogenes* are shown in Figure 8d. The liver showed normal hepatic parenchyma. The brain showed normal cerebral tissue with the presence of round cells infiltration within the meninges. Normal splenic histomorphology with a preserved white pulp lymphoid arrangement (germinal centers, central arteriole, marginal, and mantle zone) and preserved red pulp, including sinusoids, reticular fibers network, and the supported lymphoid cells beside normal megakaryocytes, supported the stroma and capsule. The red pulp showed moderate infiltration of mature and immature lymphocytes, as well as other inflammatory cells.

## 4. Discussion

*Listeria monocytogenes* is an opportunistic foodborne pathogen that can infect a wide range of farm animals including rabbits resulting in life-threatening listeriosis [10,12]. The challenges associated with *L. monocytogenes* ubiquity renders it a dangerous ongoing concern in animal production and food safety because of growth depression and great economic losses [10]. Indiscriminate therapeutic application of antibiotics for treating *L. monocytogenes* disrupts the host’s normal microbiotic balance and leads to the development of antimicrobial-resistant micro-organisms. Thus, an awareness of excessive antibiotics use can encourage the development of alternative safe ways, particularly, on the prophylaxis level.

Among these safe alternatives, we claimed that MSP could block the pathogen attachment or invasion of epithelial cells and consequently improve the rabbits’ growth performance and confer protection against *L. monocytogenes* infection. Especially in rabbits, mechanisms of how MSP modulate their tight junctions and immunity and counteract listeriosis are not fully understood until now. In the current study, use of MSP at a level of 1 × 10^8^ CFU/kg diet during the growing period contributed to the highest growth performance parameters and good health conditions of rabbits, suggesting the growth-promoting role of selected MSP. In accordance, supplementing rabbits’ diets with *B. subtilis* improved growth performance, immune organs’ indices, intestinal homeostasis, innate immune response, and disease resistance [43]. Moreover, [44,45,46] found an enhancement in growth performance after using various probiotic compositions. The previous authors attributed the improved growth performance to an increase in the digestive enzymes’ activities coupled with other changes, such as the maintenance of gastrointestinal tract (GIT) beneficial microbiota and a decrease in the production of ammonia. Besides, *Bacillus* species could produce extracellular enzymes and some necessary nutrients and provide essential growth factors to boost animal growth [47]. Similarly, greater BWG and lower FCR were observed in weaning rabbits fed with dietary *L. acidophilus* alone or a combination of *B. subtilis* and *L. acidophilus* [48]. These findings could result from greater nitrogen retention and nutrient digestibility in the *L. acidophilus-* and *B. subtilis-*supplemented rabbits [48,49]. Interestingly, the suppressed growth performance parameters induced after *L. monocytogenes* experimental infection were compensated in groups supplemented with MSP, suggesting their potential role in attenuating *L. monocytogenes* infection in rabbits. In the same line, *L. monocytogenes* infection impaired the growth performance parameters of rabbits; however, *L. acidophilus* administration recovered the final BWG of rabbits [50]. In this context, the growth-promoting activity of MSP may be increased from competing with pathogenic microbes in the gut and augmenting the immune system, thereby leading to higher resistance to dangerous infectious agents [51,52]. Additionally, the probiotic function mechanisms were heterogeneous, complex, and specific to each probiotic strain. They comprise pathogens’ competitive exclusion [53], ability to colonize the intestine [54], improvement in intestinal barrier functions by upregulating the TJP, and mucin expression, along with immune system regulation [55].

The digestion end products of dietary ingredients in the small intestine are principally absorbed through nutrient transporters that play a crucial role in nutrient absorption and feed utilization [40,56]. The absorption of monosaccharides in the intestine is crucial for the homeostasis of energy. *SGLT-1* and *GLUT-2* engage in the intestinal absorption of monosaccharides and their concentrations, and, consequently, control monosaccharide uptake in the small intestine. *SGLT-1* is specified in the apical membrane and it mediates glucose uptake from the intestinal lumen through the brush-border membrane into the intestinal enterocytes [57,58], while *GLUT-2* is the primary fructose and glucose transporter in the basolateral membrane [59,60,61]*. FABP-2* is implicated in the trafficking of intracellular free fatty acids and eventually enhances intestinal nutrient absorption [62]. Moreover, *CAT-1* can transport cationic amino acids (e.g., arginine, histidine, and lysine) from enterocytes to the vascular supply or vice versa. In this respect, probiotic strains have been previously shown to improve nutrient absorption [63]; however, the mechanism by which they can affect nutrient transporters at the molecular level needs further investigation. Herein, the long-term administration of MSP upregulated *SGLT-1*, *GLUT-2*, *CAT1,* and *FABP-2* genes associated with nutrient transportation even after *L. monocytogenes* experimental infection. Similarly, the relative gene expression of glucose and protein transporters showed a significant (*p* < 0.05) upregulation after feeding on multi-strain probiotics in broiler chickens [64].

The intestinal microbiota could greatly affect the host’s gut health via modulating intestinal pH, transporter gene expression, and mucosal immunity [65]. Probiotics can modify the composition of microbial species in the host gut by sustaining the balance and suppressing the growth of pathogenic bacteria [66]. The data of the present study revealed that supplementation of MSP at the dose of 1 × 10^8^ CFU/kg diet shifted the ileal and cecal microbiome composition of growing rabbits at 60 days of age towards the beneficial bacteria when compared to the control group. In contrast, the abundance of Enterobacteriaceae opportunistic pathogens was decreased after MSP supplementation, especially at higher doses. In accordance, dietary probiotics increased the numbers of beneficial bacterial organisms in the rabbits GIT than the pathogenic ones [67]. Moreover, feeding on probiotics increased the populations of Lactobacillus and Bifidobacterium species in the gut of rabbits [40,68]. The proposed probiotics mechanism of the intestinal microbiota modulation can result from reducing the oxygen or intestinal pH in the digestive tract [69]. Moreover, they could improve the intestinal tract habitat to antagonize the proliferation of pathogenic bacteria and therefore optimize the intestinal flora structure [70]. Additionally, an increase of the cecal lactobacilli counts in the rabbits supplemented with *L. acidophilus* led to higher concentrations of cecal acetic acids and total volatile fatty acids and decreased intestinal coliform colonization [48,71]. Moreover, rabbits fed *B. subtilis*/*L. acidophilus*-supplemented diets showed greater intestinal lactobacilli counts than those fed diets supplemented with *B. subtilis* alone [48]. This could be explained by the increased activities of lactobacilli after co-culturing with Bacillus species by the stimulating effect of the latter one on the biosynthesis of lactobacillus strains [72]. On the other hand, treatment with antibiotics affects not only the target bacterial microorganisms, but also the intestinal microbial communities. It has been shown that antibiotics exhibited long-lasting alterations in the intestinal microbiota, which is associated with disease occurrence [73]. Notably, after infection with *L. monocytogenes* and treatment with enrofloxacin, all counted bacterial populations were decreased, while early and continuous MSP supplementation after infection enhanced beneficial bacterial communities and decreased the colonization of harmful ones in the cecum and ileum, which are in agreement with previous studies [74,75]. Herein, reducing gut microbial damage after antibiotics treatment can be attributed to their wide antibacterial action that does not distinguish between types of bacteria [76,77]. Meanwhile, the beneficial effects of MSP on gut microbiota could be likely mediated through competitive exclusion, which mainly results from lowering luminal pH, thereby causing direct inhibition of enteric pathogens and the secretion of bactericidal proteins [78].

Cytokines are known to have a key regulatory impact in monitoring the intestinal inflammatory response. Probiotic bacteria exert immunostimulatory and immunoregulatory activities, and thus they can be extensively applied in the treatment of numerous diseases [79,80]. The beneficial effects of probiotics on the inflammatory process have been documented [54,81] and they are mainly due to their modulatory functions through the induction of various kinds of cytokines in gut-associated lymphoid tissues [80]. This was evidenced by the reduction of pro-inflammatory cytokine expression and boosting the anti-inflammatory ones. Similarly, *Lactobacillus* species triggered the immune system by boosting the expression of anti-inflammatory cytokine genes, *IFN-γ* and *IL-10* [82,83], or by preventing the expression of the pro-inflammatory cytokine genes, *TNF-α*, *IL-6,* and *IL-8* [84,85]. Additionally, the probiotics immunoregulatory effect can be related to *IL-10* production, which blunts the excessive inflammatory response [86]. On the other hand, the invasion of bacterial pathogens into intestinal epithelial cells triggers the gastrointestinal immune cells to produce cytokines, which stimulate immune responses against pathogens [87]. In this context, *L. monocytogenes* could upregulate *TNF-α* and *IL-6* genes, thereby leading to an increase in the permeability of the intestinal epithelium [88]. Stimulation of pro-inflammatory cytokines has been well studied in rabbits in response to *L.* *monocytogenes* [11]. *IL-1β* is a key pro-inflammatory cytokine that stimulates its own expression and the expression of other pro-inflammatory cytokines and chemokines, which in turn recruit inflammatory responses and trigger the induction of antimicrobial cells [54]. As anticipated in our study, elevated expression levels of pro-inflammatory cytokines (*TNF-α*, *IL-6*, *IL-8* and *IL-1β*) genes were noted in rabbits fed a basal diet and experimentally infected with *L. monocytogenes* compared with those experimentally infected and treated with either enrofloxacin or probiotics. Similarly, probiotics could blunt intestinal inflammation [89] through TNF-α inhibitory metabolites production and nuclear factor-kB signaling inhibition in the enterocytes [90]. Regarding *IFNγ*, its initial production is a crucial step for producing an immune response and regulating *L. monocytogenes* infection [91]. Moreover, *IL-10* has a predominantly antagonistic impact on inflammation, besides its crucial role in depressing the inflammatory and immune responses [92]. In the current study, oral administration of MSP induced the production of *IFNγ*, which promoted effective *L. monocytogenes* clearance and upregulated *IL-10* and *TGFβ* genes that suppress excessive inflammation and maintain intestinal immune homeostasis. The robust inflammatory response in the *L. monocytogenes* experimentally-infected group was effectively counteracted by MSP administration over the whole rearing period (prophylactic and therapeutic), thereby indicating their strong anti-inflammatory properties. In previous studies, higher transcript levels of *IFN-γ* genes were detected after *B. subtilis* pre-treatment, even after *Citrobacter rodentium* infection [93,94]. In accordance, *Lactobacillus* species triggered pathogen clearance and suppressed intestinal inflammation via stimulating the anti-inflammatory cytokines (*IL-10* and *TGFβ*) production [95,96]. On the other hand, supplementation with antibiotic growth promoters seemed to relatively downregulate the expression of inflammatory cytokine genes in the intestine after infection with live pathogens [97]. In a separate study, broilers fed a bacitracin-supplemented diet and received lipopolysaccharide (LPS) had reduced intestinal levels of inflammatory cytokines compared with bacitracin-unsupplemented and LPS-received controls [98]. Moreover, pathogen-infected chickens fed an antibiotics-supplemented diet had decreased *IL-2*, *IL-8*, *L-1β,* and *IL-6* transcript levels [97,99].

A key function of the intestinal epithelium is to form a natural barrier, which hinders pathogens and toxic constituents from entering the mucosa and coming into contact with the immune system, thereby ensuring intestinal homeostasis [100,101]. Intestinal tight junctions and their related proteins, including CLDN, zonula occludens (ZO) and occludin, are the main factors that stimulate tight junction barrier formation [102,103]. Previous reports have described their differing effects on intestinal permeability [102,104]. It has been demonstrated that intestinal microbiota efficiently contributed to intestinal immune system development, epithelial barrier augmentation, and pathogen colonization restriction [8]. Nevertheless, the intestinal microbiota can be modified by many causes and diseases involving dietary changes, stress, antibiotics, and infection [105], leading to an imbalance in intestinal homeostasis. During weaning, the decrease in the gene expression of TJP; *ZO-1*, *CLDN-1*, and occludin, could lead to barrier integrity impairment. In this context, probiotics have a diverse mechanism of action to enhance the function of the intestinal barrier and sustain homeostasis, and thus it may counteract the weaning stress. Herein, higher expression levels of *ZO-1*, *CLDN-1*, occludin, *MUC-2,* and *JAM-2* in the intestine of rabbits described that MSP, especially at higher doses, enhanced TJPs. In accordance, probiotics could enhance gut barrier function by increasing the expression of TJP genes [106,107,108]. Moreover, some *Lactobacillus* species, such as *L. plantarum,* abated barrier disruption via upregulation of TJPs [109,110]. In this study, relative TJP transcripts were significantly reduced after *L. monocytogenes* infection. However, after supplementing MSP during the whole rearing period (prophylactic and therapeutic), these reductions were abrogated. This could be attributed to the beneficial roles of MSP in fortifying the intestinal epithelial resistance to pathogens by sustaining TJP abundance. A previous study utilizing different probiotic strains described parallel in-vivo and in-vitro results [111]. It has been asserted that lactic acid bacteria (LAB) can restore the intestinal epithelial barrier damage generated by pathogenic infection [109]. A recent report found that lactobacilli ameliorated the intestinal barrier damage induced by *Salmonella* species [112]. Moreover, [113] described that lactobacilli probiotics prevented *L. monocytogenes-*induced intestinal permeability through the preservation of the cell junctional architecture of *CLDN-1*, occludin, and E-cadherin. Additionally, *L. plantarum* had protective effects on the intestinal barrier by rearranging TJPs (*ZO-1*, *CLDN-1*, and occludin) disturbed by *Escherichia coli* (*E. coli*) and accordingly ameliorated the barrier function [113,114]. Another in-vitro study described that *L. rhamnosus* and *L. fermentum* significantly improved *E. coli*-disturbed TJP (Occludin, *ZO-1*, cingulin-1, and *CLDN-1*) [115]. Notably, maintaining tight junction integrity in rabbits that received MSP during the rearing period was in line with our results of downregulating the pro-inflammatory cytokine genes (*IL-6*, *IL-8,* and *TNFa*). Aside from their important roles in immunity, cytokines were also demonstrated to affect tight junction integrity as pro-inflammatory cytokines could induce tight junction disruption. Commensal bacteria such as *Lactobacillus* and *Bifidobacterium* species in intestinal microflora could improve intestinal barrier functions and regulate cytokines secretion [84]. Similarly, *L. plantarum* pre-treatment downregulated the pro-inflammatory cytokine genes (*IL-6*, *IL-8,* and *TNF-α*) and alleviated the reduction in TJP (*CLDN-1*, occludin, and *ZO-1*) caused by *E. coli* [54]. Furthermore, the intestinal barrier is reinforced by a glycosylated mucin-rich layer secreted by goblet cells. In view of this, probiotics have been demonstrated to strengthen the integrity of intestinal barriers by increasing the number of goblet cells that reinforce the mucus layer [116]. Previous in-vitro studies described that many *Lactobacillus* species were proved to increase the expression of intestinal mucin [117,118,119]. Moreover, lactobacilli probiotics triggered MUC secretion and thus fortified the mucus barrier and limited *L. monocytogenes-*induced MUC-2 loss and apoptotic responses [113]. Although antibiotics can combat pathogenic agents, their application exhibited several adverse effects, such as intestinal barrier dysfunction [40]. This is supported by our results, which evidence that treatment with enrofloxacin decreased the expression of TJP genes. Similarly, [120] verified that antibiotics reduced the expression of TJP genes and increased intestinal paracellular permeability, suggesting their negative impact on intestinal tight junction barriers. Herein, the impairment of intestinal barrier functions is associated with dysbiosis of intestinal microbiota and the decrease in the expression of *ZO-1*, *CLDN-1*, and occludin genes post-treatment with enrofloxacin.

Regarding the infection with *L. monocytogenes,* it enters the host via the intestines and then infects the spleen, liver, and immune cells, where they proliferate until attacked by the host’s immune system [121]. As an important weapon to struggle against infectious diseases, antibiotics have been employed to cure bacterial infections for several years. Even though antibiotics bring considerable advantages for the hosts, they produce a variety of grievous adverse concerns. Among them, infections with multidrug-resistant pathogens might be the most dangerous one [122]. From this view, the potential effects of probiotic bacteria have been intended as a rational attempt for counteracting the intestinal pathogens [47,123]. Regarding farmed rabbits, there is limited data concerning the prevention or control of *L. monocytogenes* infection by probiotics. Our results proved that MSP administration during the rearing period greatly reduced the intestinal counts of *L. monocytogenes*. In accordance, [113] described that the lactobacilli probiotics were able to co-aggregate with *L. monocytogenes* and inhabit the membrane expressed epithelial Hsp60 receptor sites on epithelial cells to competitively eliminate it. There are several mechanisms underlying the effect of probiotic strains on the inhibition of gut translocation of bacteria to other organs [124]. It is proposed that probiotics may compete with pathogens for adhesion sites, prevent pathogen-induced disruption of epithelial integrity, and modulate the immune system, thereby conferring resistance to *L. monocytogenes* [23]. Besides, LAB also produced microbicidal substances that have a great impact on the gastric and intestinal pathogens and other microbes that compete for cell surfaces and mucin binding sites. Current in-vitro findings revealed that *L. acidophilus*, *L. plantarum*, and *E. faecium,* or their secondary metabolites, prevented the formation of *L. monocytogenes* biofilm [92,125,126]. Additionally, the reduced consequences of the probiotic on *L. monocytogenes* loads were endorsed by recent results in rabbits [11]. After infection with *L. monocytogenes*, significant histological changes were detected in rabbits’ livers, brains, and spleens with a picture of septicemia (positive control group). Similar findings were previously detected in the organs of rabbits infected with *L. monocytogenes* [127]. Administration of MSP during the whole rearing period attenuated the translocation of *L. monocytogenes* to other organs, as evidenced by restoring the normal histopathological architecture of rabbits’ livers, brains, and spleens. Similarly, significant improvements were found in the histological pictures of rabbits` tissues after administration of *Lactobacillus acidophilus* [128]. This could be attributed to the effective roles of MSP, which was proven in our results, on enhancing the immunity of rabbits against infection with intestinal pathogens and strengthening the intestinal barriers with a consequent restriction of pathogen spread to different organs. 

## 5. Conclusions

Taken together, our findings suggested the favorable outcomes of multi-strain probiotics during the whole rearing period on rabbits’ growth, immune response, and intestinal barriers, as evidenced by modulating the expression of cytokines and tight-junction-protein-related genes. Additionally, administration of MSP for rabbits infected with *L. monocytogenes* attenuated the severity of clinical signs, PM lesions, and pathogen localization or translocation by decreasing its load in the cecum of rabbits, thereby downregulating the pro-inflammatory cytokines and subsiding the excessive inflammatory response. Hence, our results recommend the application of multi-strain probiotics, as an alternative to antibiotics, to offer protection for rabbits against *L. monocytogenes* infection.

## Figures and Tables

**Table 1:** Feed ingredients’ levels and nutrient composition of the control experiment diet.

Ingredient	%
Yellow corn	10
Barley grains	16.3
Soybean meal, 44%	15.70
Berseem hay	33.2
Wheat bran	19
Molasses	3.00
Premix *	0.3
Calcium dibasic phosphate	1.5
Common salt	0.5
Antitoxin	0.3
Anticoccidial	0.2
Nutrient level	
DE, Kcal/Kg	2555.60
CP	16.33
EE	2.33
CF	12.56
Ca	1.09
Phosphorus	0.59

* Premix: each 5 kg is composed of vitamins, D3: 3,000,000 IU, E: 3300 mg, A: 1850,000 IU, B1: 220 mg, B2: 700 mg, B12: 2.5 mg, and B6: 300 mg; calcium antothenate: 2500 mg; nicotinic acid: 4600 mg; choline: 10,000 mg; Magnesium: 100 g; Cu: 4000 mg; Mn: 10,000 mg; I: 300 mg; Co: 25 mg; Fe: 10,000 mg; Se: 25 mg, and Zn: 12,000 mg; DE: digestible energy; CP: crude protein; EE: ether extract; CF: crude fiber and Ca: calcium.

**Table 3:** Effect of various levels of multi-strain probiotics supplementation on growth performance attributes of rabbits at the end of the growing period (60 d).

Parameter	Experimental Groups				*p* Value	SEM
	Control	MSPI	MSPII	MSPIII		
BW, g	1818 ^d^	2006 ^c^	2147 ^b^	2176 ^a^	<0.02	12.14
BWG, g	1086 ^d^	1204 ^c^	1344 ^b^	1372 ^a^	<0.001	13.16
FI, g	2700 ^c^	2991 ^b^	3084 ^a^	2942 ^b^	0.03	14.83
FCR	2.49 ^a^	2.48 ^a^	2.29 ^b^	2.14 ^c^	0.01	<0.001

BW: body weight; BWG: body weight gain; FI: feed intake; FCR: feed conversion ratio; MSP: multi-strain probiotics, including *Lactobacillus acidophilus* NBIMCC 8242, *Bacillus subtilis* DSM 17,299, and *Enterococcus faecium* NBIMCC 8270. Control: rabbits fed a basal diet; MSPI, II and III: rabbits fed a basal diet supplemented with MSP at the levels of 1 × 10^6^, 1 × 10^7^ and 1 × 10^8^ CFU/kg, respectively. SEM: standard error of the mean. Means with different superscripts (a–d) within the same row differ significantly (*p* < 0.05).

**Table 4:** Effect of multi-strain probiotics and enrofloxacin supplementation on growth performance attributes of experimentally-infected rabbits at the end of finishing period (90 d).

Parameter	Experimental Groups				*p* Value	SEM
	PC	PC + ENR	MSPP	MSPTT		
BW, g	2110 ^d^	2664 ^b^	2431^c^	2742 ^a^	<0.001	34.93
BWG, g	1308 ^d^	1862 ^b^	1627 ^c^	1938 ^a^	<0.001	33.34
FI, g	4832 ^d^	5756 ^a^	5214 ^c^	5654 ^b^	0.04	15.37
FCR	3.69 ^a^	3.09 ^c^	3.20 ^b^	2.92 ^d^	0.02	0.09
Mortality %	24.00 ^d^	7.80 ^b^	20.00 ^c^	6.00 ^a^	<0.001	0.07

BW: body weight; BWG: body weight gain; FI: feed intake; FCR: feed conversion ratio; MSP: multi-strain probiotics, including *Lactobacillus acidophilus* NBIMCC 8242, *Bacillus subtilis* DSM 17,299, and *Enterococcus faecium* NBIMCC 8270. PC: positive control (rabbits fed a basal diet and experimentally infected with *L. monocytogenes*); PC + ENR: rabbits fed a basal diet, experimentally infected with *L. monocytogenes* and treated with enrofloxacin; MSPP: rabbits fed a basal diet supplemented with MSP at the level of 1 × 10^8^ CFU/kg from 30 to 60 days of age (prophylactic) and experimentally infected with *L. monocytogenes*; MSPTT: rabbits fed a basal diet supplemented with MSP at the level of 1 × 10^8^ CFU/kg from 30 to 90 days of age (prophylactic and therapeutic) and experimentally infected with *L. monocytogenes*. Rabbits were orally administered with *L. monocytogenes* at the concentration of 10^7^ CFU/mL at 60 days of age. SEM: standard error of the mean. Means with different superscripts (a–d) within the same row differ significantly (*p* < 0.05).

## Data Availability

The data presented in this study are available on request from the corresponding author.
